# Target-biology and interactome-derived signatures predict target-level associations with safety-related drug attrition

**DOI:** 10.1038/s41598-026-65633-y

**Published:** 2026-08-24

**Authors:** Jordan Breffle, Alex Jones, Susan D. Ghiassian

**Affiliations:** Departments of Data Science and Network Medicine, Scipher Medicine Corporation, Waltham, MA USA

**Keywords:** Target safety, Toxicity prediction, Network medicine, Human interactome, Machine learning, Computational biology and bioinformatics, Drug discovery

## Abstract

Clinical drug development suffers from high rates of toxicity-related failure despite the use of compound-centric preclinical safety screening, with approximately one-third of all clinical failures attributable to safety concerns. An ability to prioritize early-stage drug development programs toward those with a lower probability of causing clinical toxicity would improve drug development success rates. Here, we propose a target-centric framework that integrates network medicine principles with target biology features to predict operational target-level labels associated with safety-related drug attrition. From a set of 3,696 drugs with widely launched or safety-related termination outcomes, we curated 541 non-overlapping target labels, comprising 302 safety-liability-associated targets and 239 widely launched-associated targets. We engineered target-level features encoding both biological properties and human interactome (HI) topology and trained a gradient boosting classifier to predict the safety-liability-associated label. The model achieved a held-out test ROC AUC of 0.712. These results suggest that target biology and interactome context contain signal associated with safety-related clinical attrition and may support early target prioritization when used alongside compound-centric safety assessments.

**Supplementary Information:**

The online version contains supplementary material available at 10.1038/s41598-026-65633-y.

## Introduction

Current drug development practices result in the approval of only ~10–15% of drugs that enter human trials, with approximately 30% of all clinical failures due to unacceptable toxicity^1,2^. Improved in silico methods for predicting drug toxicity could significantly improve the success rate of clinical trials by prioritizing drugs that are less likely to fail due to toxicity, thereby redirecting drug-development resources to candidates with higher likelihoods of success.

Current approaches for evaluating toxicity remain predominantly compound-centric, focusing on chemical structure-based liabilities such as hERG channel blockade, reactive metabolite formation, and promiscuous receptor binding, which are tested through preclinical in vitro safety panels^3^. While these screens can identify off-target risks associated with a specific compound, they fail to provide insight into whether the intended on-target mechanism itself poses toxicity concerns. Accumulating evidence from human genetics and clinical safety data suggests that intrinsic target biology is a significant determinant of therapeutic tolerability independent of compound structure^4–6^. However, safety still accounts for a substantial fraction of clinical failures despite widespread genetics-guided target prioritization, implying that genetic evidence alone and target-by-target analysis often fail to capture mechanism-based risk encoded in a target’s broader interaction network. This mechanistic disconnect motivates the development of complementary target-centric safety-liability models that can flag targets whose biological and network context resembles targets associated with prior safety-related attrition.

Network medicine offers a promising framework for such target-focused safety assessment. In this paradigm, gene products are represented as nodes in a graph and their physical interactions are represented as edges^7,8^. The human interactome (HI) is the resulting genome-scale network of human gene products and their interactions. Analysis of the HI has revealed that drug targets with higher centrality measure values or with closer proximity to essential cellular machinery are more likely to be associated with adverse events^9,10^. Early studies in yeast revealed a centrality‒lethality principle, wherein highly connected hub nodes are enriched for essential genes whose deletion is lethal^11^, a principle that extends to humans^12,13^. Similarly, cancer genes are associated with higher than average HI degrees and are more likely to correspond to hub nodes^12,14^, and disease-associated genes have been shown to fall within distinct network neighborhoods (*disease modules*)^7^. Perturbations within disease modules can give rise to disease phenotypes, and the network proximity of a drug target to disease modules and to proteins implicated in adverse drug reactions has been associated with increased risk of clinical side effects^7,15,16^. These observations suggest that integrating network topology with tissue expression patterns and other target-level properties could yield reproducible target-level safety predictions.

Here, we present a network medicine approach for target-level safety-liability prediction, building a model that assigns each interactome node a score reflecting similarity to targets associated with safety-related drug attrition labels. We curated operational target-level labels from drug records with widely launched (“safe”) or safety-related termination (“toxic”) outcomes, engineered features encoding network proximity to essential and disease-associated genes, tissue expression patterns, network centrality, and other biological properties, and then trained a gradient boosting classifier using fivefold cross-validation, achieving an independent test-set ROC AUC of 0.712. We then identified pathway enrichment differences between the safe and toxic targets, finding that toxic targets are enriched for a greater number of pathways and preferentially enriched for cancer-related pathways.

## Results

### Overview of the drug target toxicity prediction framework

**Fig. 1:**
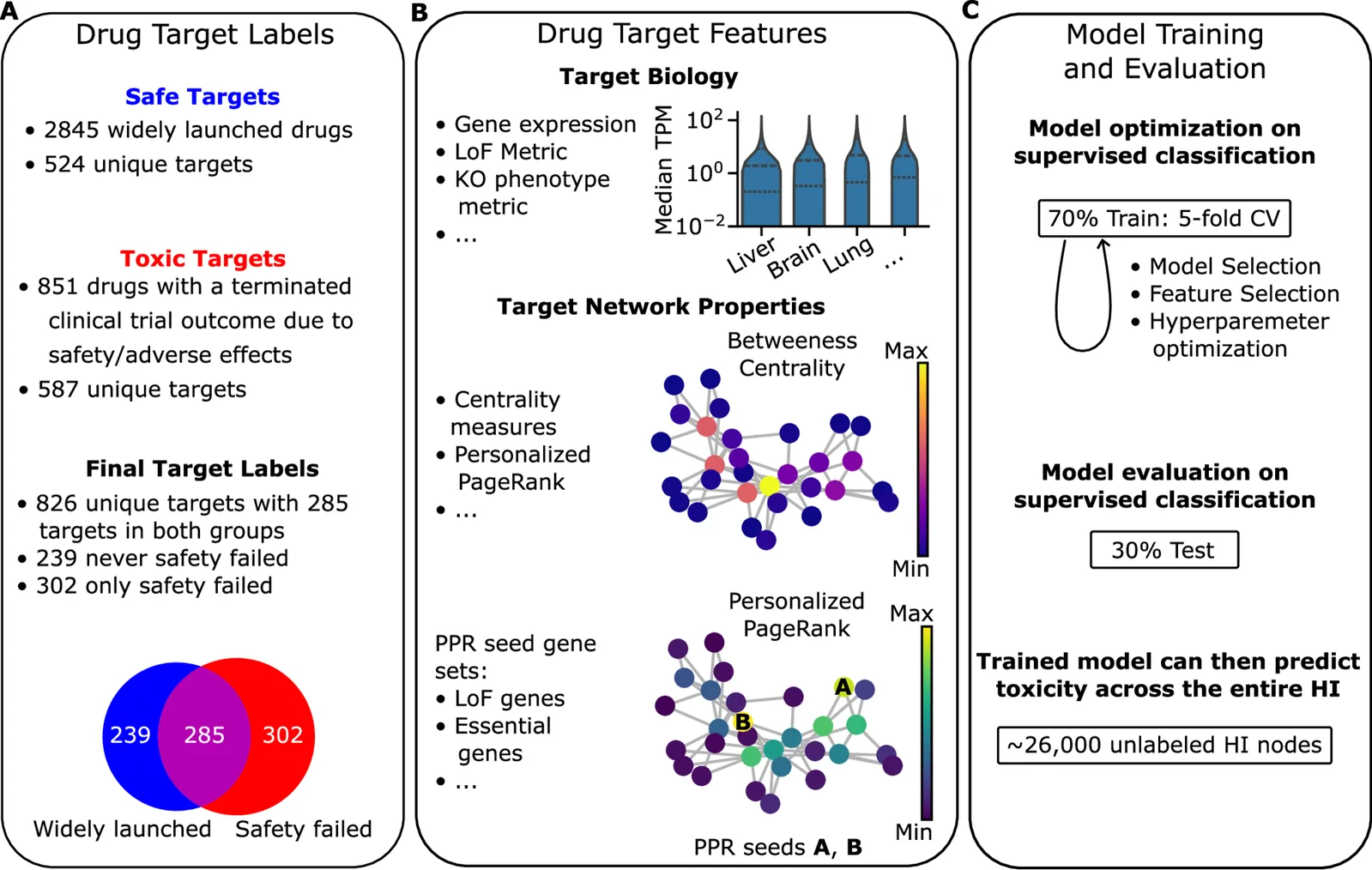
Illustration of methods and target labeling. **A**: Summary of target toxicity labeling. Top, description of the source of drug safety outcome derived target labels. Bottom, Venn diagram showing the number of targets of drugs that are widely launched (“safe” targets) and safety failed (“toxic” targets). **B**: Summary of target feature engineering. Top, example list of target biology-based features, including an illustration of distributions of expression levels across a subset of tissues. Center, example list of target features derived from network properties, including an example graph illustrating betweenness centrality. Bottom, example list of gene sets used as seeds for personalized PageRank (PPR) based features, including an example graph demonstrating PPR values. **C**: Overview of model training and evaluation. Top, illustration of the model optimization process using 70% of the labeled targets. Center, illustration of model evaluation on the 30% of labeled targets held out as a test set. Bottom, illustration of how the model trained on the labeled targets can then be applied to predict toxicity across the entire interactome.

Our toxicity prediction approach leverages a gradient boosting classifier to predict drug target toxicity from a feature vector that includes both target biology and target network properties (Fig. 1). We used the Citeline drug database^17^ to curate two operational target-level label groups from drug records with distinct clinical-development outcomes (Fig. 1A). The widely launched-associated group consisted of targets annotated for drugs labeled “Widely Launched” in Citeline Pharmaprojects, yielding 524 unique targets from 2,845 unique drugs. The safety-liability-associated group consisted of targets annotated for drugs in trials labeled “Terminated, Safety/adverse effects” in Citeline Trialtrove, yielding 587 unique targets from 851 unique drugs. To reduce label ambiguity, we excluded 285 targets that appeared in both groups. The resulting non-overlapping dataset contained 239 widely launched-associated targets and 302 safety-liability-associated targets. For label brevity, we refer to these two sets of targets in figures as “safe” and “toxic”, respectively.

**Fig. 2:**
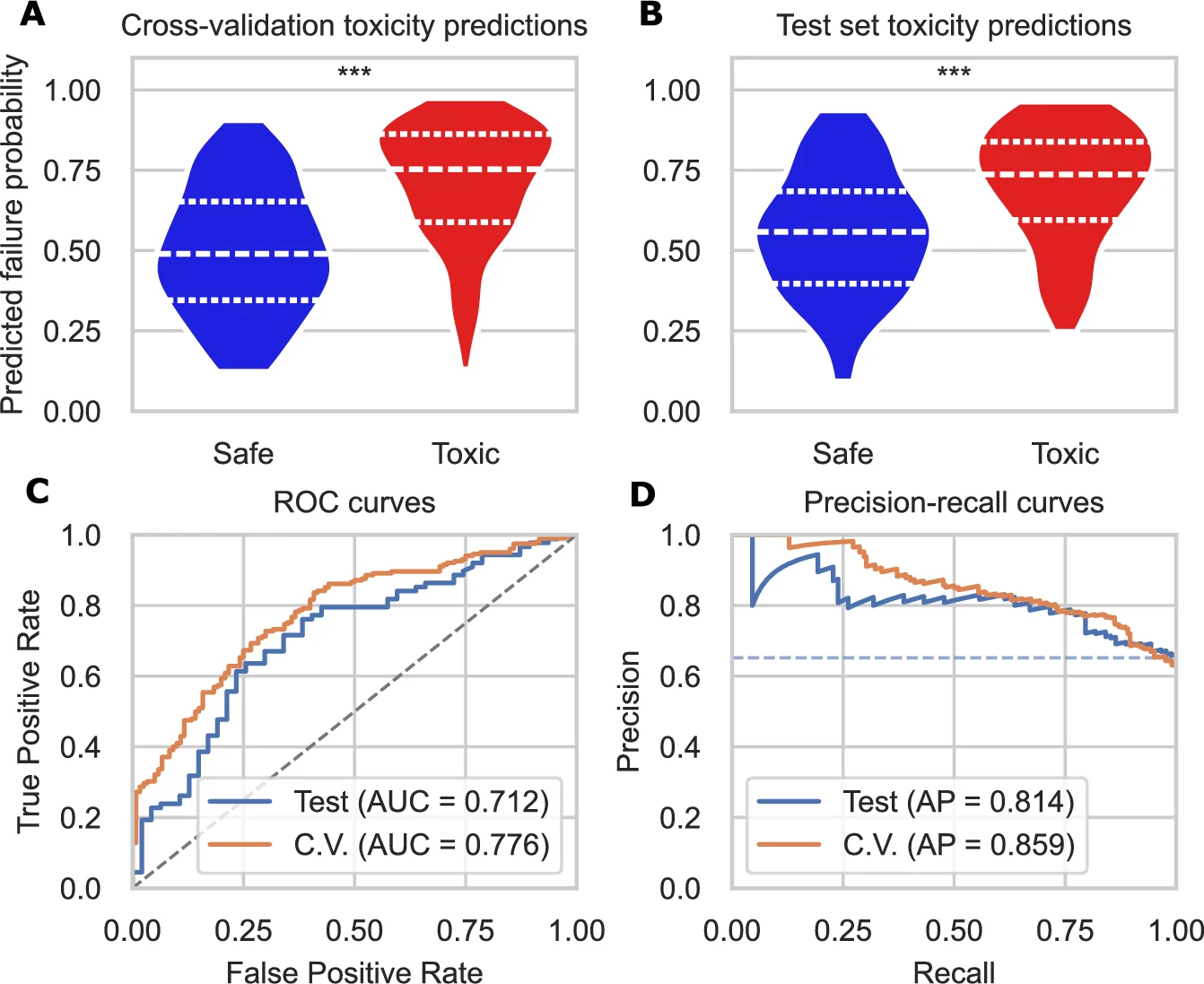
Model performance. **A**: Distributions of the predicted probabilities of target toxicity for the cross-validation training data. **B**: Same as **A** but for the held-out test data. **C**: Receiver operating characteristic (ROC) curves for the cross-validation training data predictions and the test data. **D**: Precision-recall curves for the cross-validation training data and the test data. Abbreviations: AP, average precision; AUC, area under the curve. *** for *p<0.001*, Mann‒Whitney U Test.

We integrated multiple public databases to derive target-level features related to each target’s biological properties as well as network properties in the HI (Fig. 1B, Fig. S1, Table S1). Using the target-level features and operational target labels derived from clinical-development outcomes, we trained a machine learning classifier to predict drug target toxicity (Fig. 1C, Fig. 2). We split the labeled data into a 70/30 training/testing split and used cross-validation performance on the training set to perform model selection, feature selection, and hyperparameter optimization. After locking the model based on performance optimization on the training set, we evaluated performance on the test set.

### Target features

To build our toxicity prediction model, we collected and engineered target-level features that describe various properties of each target in the HI, ranging from network topology to tissue expression patterns and other biological properties (see Methods). For the features evaluated as possible inputs to the optimized model, we observed that many showed statistically significant but generally modest differences between widely launched-associated and safety-liability-associated targets (Table S1, Fig. S1).

We compared the median TPM (transcripts per million) gene expression levels from the GTEx database between the two groups across the 54 tissues included in the database and performed principal component analysis (PCA) to identify the principal components (PCs) of the target cross-tissue expression levels (Table S1, Fig. S1). We found that several tissue-expression features differed between the two target-label groups. In particular, safety-liability-associated targets showed higher expression in immune-enriched or immune-active tissues, including whole blood, spleen, lung, and lymphoid aggregates in the terminal ileum (Table S1, Fig. S1). These associations may reflect enrichment of safety-liability-associated targets in immune, inflammatory, hematologic, mucosal immune, and barrier-tissue biological contexts. However, these tissue-expression differences are associative and do not identify the mechanism of safety-related attrition for any individual drug-target pair. Cross-tissue gene-expression principal components showed only modest separation between the two groups, with two of the top five PCs showing statistically significant differences (Table S1, Fig. S1).

We used the HI to evaluate whether the network topology of targets was predictive of target toxicity (Table S1, Fig. S1). We ran the PPR algorithm with different sets of seeds, defined by different criteria of important or dangerous genes, including essential genes (BAGEL)^18^, haploinsufficient genes (ClinGen)^19^, and oncogenes (GenCC Strong Oncology and IntOGen Pan-cancer recurrent)^20,21^. For all the PPR values derived from the different sets of seed genes, safety-liability-associated targets had significantly greater values, indicating that they were more proximal topologically to the set of seed genes than were widely launched-associated targets (Table S1, Fig. S1). This pattern is consistent with the hypothesis that targets located near essential, haploinsufficient, oncogenic, or disease-associated regions of the HI may carry elevated safety liability when perturbed. These associations support the biological plausibility of the label groups but do not establish causality for individual drug failures.

In addition to gene expression and network-derived features, we incorporated features related to target safety and druggability from the Open Targets database (Table S1, Fig. S1)^22^. To avoid data leakage, we restricted these Open Targets features to annotations of intrinsic target biology and tractability that were not derived from clinical safety outcomes. We observed trends for these features similar to the expression and HI-based features, wherein safety-liability-associated targets showed modest but significant differences in features plausibly associated with safety-liability.

### Evaluation of model performance

From the target-level features, we trained a machine learning model to predict the operational safety-liability-associated target labels (Fig. 2). We used cross-validation performance to perform model selection, feature selection, and hyperparameter optimization by optimizing for the area under the curve (AUC) of the ROC curve.

The optimized model performs significantly better than chance on both the cross-validated training data (Fig. 2A, $p=1.25\times {10}^{-16},$ U-statistic $=5.43\times {10}^{3}$) and the held-out test data (Fig. 2B, $p=5.11\times {10}^{-5},$ U-statistic $=1.19\times {10}^{3}$). The model performance generalizes well to the test set as indicated by the AUC (Fig. 2C) and average precision (AP; Fig. 2D) values calculated from the predictions for the cross-validated training data (AUC = 0.776, AP = 0.859) and the test data (AUC = 0.712, AP = 0.814). Because the positive class was more frequent than the negative class after HI mapping, we interpreted average precision relative to the no-skill baseline equal to the positive-class prevalence in the corresponding evaluation set. To support practical prioritization, we additionally evaluated the locked model at prespecified top-ranked cutoffs and at a decision threshold selected only within the training cross-validation procedure. We set a decision threshold of 0.756 based on the threshold at which the recall was 0.5 on the cross-validation training results. At this threshold on the held-out test set, the model achieved precision = 0.812, recall = 0.442, specificity = 0.809, false-positive rate = 0.191, false-discovery rate = 0.188, and enrichment factor = 1.25. At the top 5%, 10%, and 20% of ranked targets, enrichment factors were 1.36, 1.45, and 1.24, respectively.

### Pathway enrichment analysis of safe and toxic targets

**Fig. 3:**
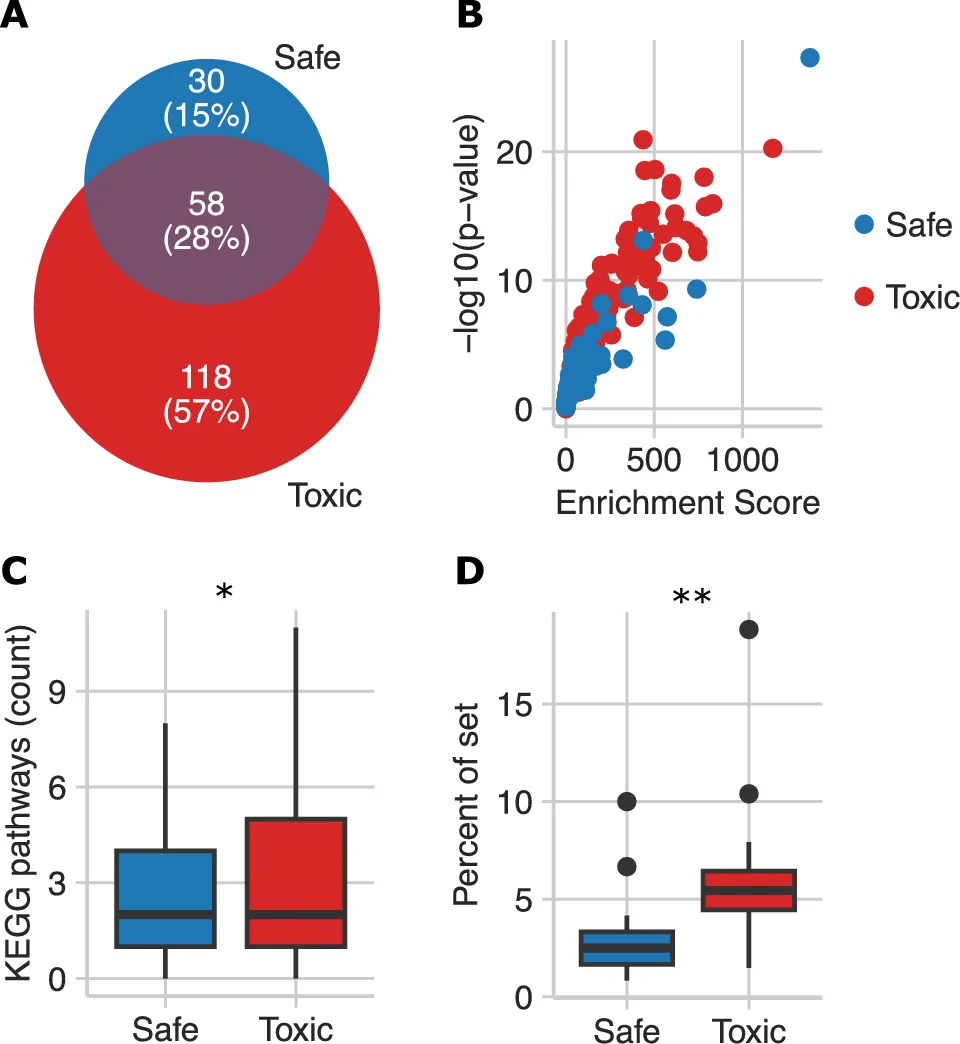
Relative to safe targets, toxic targets are enriched in more KEGG pathways and more cancer pathways. Pathway enrichment was performed using the KEGG_2021_Human gene-set library accessed through Enrichr^39,40^. **A**: Venn diagram of the number of pathways for which safe (blue) and toxic (red) targets are enriched. **B**: Scatter plot of the enrichment score and enrichment p-value for all pathways enriched with either safe or toxic targets. **C**: Box plot of the number of pathways enriched per target for safe and toxic targets. Toxic targets are enriched for significantly more pathways (Student’s t-test, *p=0.016*, *t=2.4198*). **D**: Percentage of safe and toxic targets enriched for a cancer-related pathway. Toxic targets are enriched for significantly more cancer pathways (Student’s t-test, *p=0.009*, *t=2.8021*). * for *p<0.05*, ** for *p<0.01*.

To understand the biological basis for the differences between the widely launched-associated and safety-liability-associated target groups, we performed pathway enrichment analysis to test whether safe and toxic targets showed differential pathway enrichment. We found that both safe and toxic targets were significantly enriched in many pathways (Fig. 3A-B; Table S2). We found that, relative to safe targets, toxic targets are enriched in more pathways per gene (Fig. 3C) and are more likely to be enriched in a cancer pathway (Fig. 3D). Together, these results demonstrate that safety-liability-associated targets show broader pathway enrichment than widely launched-associated targets. Relative to safe targets, a greater proportion of toxic targets contribute to a greater number of pathways.

To evaluate the relationship between these pathway enrichment results and the results of our toxicity prediction model, we analyzed the overlap between the gene seed sets used for the PPR-based features and the labeled safe and toxic targets (Fig. S2). We found that the widely launched-associated targets significantly overlapped with the two seed sets related to disease genes and haploinsufficient genes but not with the four seed sets related to essential genes or oncogenes. In contrast, the safety-liability-associated targets significantly overlapped with all six seed sets (Fig. S2). This broader overlap provides biological support for the operational label strategy by showing that safety-liability-associated targets are enriched in network neighborhoods linked to essentiality, haploinsufficiency, oncology, and disease biology.

## Discussion

Our results demonstrate that target-level labels associated with safety-related clinical attrition can be reliably predicted from a feature set combining network topology, tissue expression patterns, and other biological properties when trained on operational target labels derived from clinical outcome records. Our gradient boosting classifier achieved an ROC AUC of 0.712 and average precision of 0.814 on the held-out test data. This performance was achieved by leveraging known correlates of adverse event burden, including personalized PageRank proximity to biologically important genes, tissue expression patterns, betweenness centrality, and loss-of-function intolerance.

Our selection of candidate model features was motivated by past literature describing complementary mechanisms by which targets may incur safety liabilities. HI nodes with high centrality measure values, particularly betweenness, correspond to proteins that are more likely to act as hub nodes whose perturbation could disrupt multiple downstream pathways, a topological signature that correlates strongly with clinical adverse event profiles^9,10^. These hub nodes are enriched for essential genes and often participate in essential complex biological modules^11,13,23^. Wang et al. (2013)^9^ demonstrated that drugs targeting essential genes cause significantly more side effects, with the number of essential targets (rather than the total target count) being the primary determinant of adverse event burden. Targets with high personalized PageRank scores relative to essential genes, oncogenes, and disease-associated genes reside in functional proximity to the cellular machinery required for proliferation and survival or within critical disease modules^15,16^, explaining their elevated probability of toxicity. The breadth of tissue expression, captured by principal component features, reflects the pleiotropic involvement that amplifies on-target toxicity risk^6^. The convergence of network topology, expression patterns, and genetic constraint as primary drivers suggests that these represent complementary views of a unified biological principle: targets embedded in essential, broadly active cellular processes incur elevated safety liabilities.

To probe the biological basis of these predictions, we performed network and pathway analyses to compare safe and toxic targets. We found that toxic targets significantly overlapped with each of the gene sets used as PPR seed sets, including essential genes, oncogenes, and disease-related genes (Fig. S2), which was consistent with enrichment in proliferative and disease-relevant HI neighborhoods. In addition, pathway enrichment analysis revealed more extensive pathway enrichment among toxic targets, including differential enrichment for cancer-related pathways (Fig. 3). Taken together, these findings support the same model-level interpretation that proximity to essential, oncogenic, haploinsufficient, and disease-associated gene neighborhoods, coupled with broader pathway enrichment, distinguishes targets associated with safety-related attrition from targets associated with widely launched drugs. These results support the biological plausibility of the safety-liability-associated label group, while not proving causal toxicity mechanisms for individual targets.

Given this biological grounding, once trained to predict toxicity from labeled nodes, the model can then be applied to any node in the HI to generate genome-wide toxicity probability scores, enabling prospective target prioritization. For early-stage drug development at the novel target identification stage, safety scores can filter large candidate lists, prioritizing lower-risk entry points before significant drug development costs are incurred. For established targets with mixed clinical histories, the model’s risk estimates can facilitate indication selection by prioritizing only indications where the therapeutic benefits would be significant enough to outweigh the predicted toxicity concerns.

While the model shows strong held-out discrimination and supports the presence of target-biology signal, several limitations warrant consideration and offer avenues for future investigations. Our curated training label set reflects only targets with publicly disclosed outcomes recorded in the Citeline database, introducing a potential selection bias toward well-validated mechanisms and underrepresenting novel target classes. Because we limited our training label set to only those with unambiguous clinical safety outcomes, its size is inherently limited, which constrains model complexity, although gradient boosting has demonstrated effectiveness in other comparable toxicity modeling studies, including radiation-induced toxicity prediction and compound-centric toxicity prediction^24,25^. Additionally, therapeutic context must play a role in the consideration of toxicity tolerance. For example, a target deemed too toxic for the treatment of a chronic metabolic disease might be acceptable for an oncology therapeutic. Importantly, because our approach focused exclusively on target-level features, it cannot distinguish on-target from off-target mechanisms for individual drug failures and cannot model compound-specific liabilities such as promiscuous binding, reactive metabolites, formulation, exposure, or pharmacokinetic effects. These compound-centric toxicity concerns need to be addressed with complementary approaches, such as in vitro screening^3^ and structure-based toxicity prediction methods^26,27^, and future work could extend our toxicity prediction approach to include both target-level and compound-level features. Methodologically, gradient boosting was selected for its interpretability and robustness with small sample sizes, but alternative architectures including graph neural networks that explicitly model interactome structure^28^ or approaches that leverage both compound and target features^29^ merit exploration for future work.

Past toxicity prediction work has been primarily compound-centric, using drug-level features as model inputs and using readouts from assay-based compound-centric benchmarks such as Tox21^30^ as the endpoint for prediction. The top models using that approach include DeepTox, which reported an overall average ROC AUC of 0.846 across sub-challenges of Tox21^26^, and eToxPred, which reported an ROC AUC of 0.82 for compound-level toxicity prediction^31^. Related proteome-scale approaches have also predicted drug-protein interaction profiles across the human proteome and used those profiles to infer drug side effects, providing a framework for evaluating target-mediated safety liabilities arising not only from the intended therapeutic target but also from secondary targets and off-target interactions^32^.

Our model addresses a different point in the safety-assessment workflow. Rather than predicting the full interaction profile or side-effect profile of a particular compound, it estimates safety-liability-associated patterns for the annotated therapeutic target using target-intrinsic and interactome-derived features. The TargeTox model is most similar to our approach and uses both compound and target features to predict clinical toxicity in drug development, with a reported ROC AUC of 0.713 on held-out test data^29^. Together, these studies highlight that performance claims in toxicity prediction are strongly dependent on the endpoint, the unit of prediction, and whether the model represents the intended target alone or the broader compound-target interaction profile. They also motivate the evaluation of intended-target, drug-protein interaction, and compound-centric approaches as complementary rather than directly interchangeable.

In summary, we present a network medicine framework for predicting target-level biological associations with safety-related clinical attrition that achieves robust predictive performance through the integration of interactome topology, tissue expression patterns, and other target-level properties. This approach fills a critical gap in current approaches for drug development safety assessment, which emphasize compound structure but neglect intrinsic target biology, and provides interactome-wide safety scoring, enabling improved target prioritization. Combining target-level toxicity prediction with target-level efficacy prediction^33^ could enhance novel target discovery and target prioritization efforts.

## Methods

### Clinical safety data and target labeling

We assembled a target-level toxicity dataset from the Citeline databases Pharmaprojects and Trialtrove^17^. For safety-liability-associated targets, we extracted all drugs annotated as “Terminated, Safety/adverse effects” in the Citeline Trialtrove clinical trials database, which includes trial outcomes across all clinical trial stages, identifying 587 unique targets from 851 drugs. For widely launched-associated targets, we extracted all drugs labeled “Widely Launched” in the Citeline Pharmaprojects drug database, identifying 524 unique targets from 2,845 drugs. Drug‒target mappings were obtained from the respective Citeline databases and normalized to Entrez Gene identifiers.

These labels were designed for target-level model training and are not causal adjudications of individual drug failures. A safety-related drug termination can result from on-target pharmacology, off-target pharmacology, polypharmacology, pharmacokinetics, exposure, formulation, patient population, disease context, or other compound-specific mechanisms. Conversely, a widely launched drug does not establish that every annotated target is intrinsically safe in all therapeutic contexts. We therefore interpret the positive class as “safety-liability-associated” and the negative class as “widely launched-associated,” rather than as definitively toxic or safe targets.

To ensure high-confidence labels, we implemented a consensus strategy that excluded targets with conflicting evidence. Specifically, we removed 285 targets that appeared in both the safe and toxic sets, yielding a final set of 302 safety-liability-associated targets and 239 widely launched-associated targets (541 total labeled targets). This exclusion strategy prioritized non-overlapping operational labels over dataset size. The resulting labels distinguish targets associated only with safety-related termination records from targets associated only with widely launched drug records in our curation.

### Human interactome

We used a human interactome (HI) assembled from experimentally validated protein‒protein interactions from 21 public databases^7,34,35^, combined with experimentally confirmed binding interactions mediated by noncoding RNAs, as previously described^8^. The network comprises 26,344 nodes and 1,103,828 edges, where the nodes correspond to gene products (70.1% protein-coding, 14.6% ncRNA, and 14.5% other), and the edges indicate experimentally confirmed physical interactions between two gene products. Among the 541 labeled targets, 457 (84.5%) mapped to nodes in the HI. The targets that did not map to the HI were predominantly non-human targets related to the treatment of infectious diseases. We restricted model training to the 457 interactome-mapped targets to enable calculation of network-derived features, resulting in 290 toxic targets and 167 safe targets.

### Feature engineering

#### Feature set rationale

The feature set was designed to capture complementary and biologically interpretable dimensions of target-level safety liability. Tissue-expression features were included because perturbation of a target expressed in many healthy tissues, or in tissues vulnerable to adverse effects, may have broader physiologic consequences. Cross-tissue expression principal components were included to summarize multi-tissue expression patterns without relying only on individual tissue-level measurements. Interactome centrality features were included because highly central proteins may influence many downstream biological processes, making their perturbation more likely to have pleiotropic effects. Personalized PageRank features were included to measure network proximity to biologically relevant seed sets, including essential genes, haploinsufficient genes, cancer-related genes, and disease-associated genes. These seed sets were selected because proximity to the network neighborhoods of these gene sets may indicate greater potential for safety liability when a target is pharmacologically perturbed. Genetic-constraint and mouse-knockout features were included as orthogonal evidence about organismal tolerance to target perturbation. Target-biology and tractability features, including tissue specificity, tissue distribution, membrane localization, secretion, and cancer-driver status, were included because they capture interpretable biological properties relevant to therapeutic modulation.

#### Tissue expression features

We obtained adult human bulk tissue median transcripts per million (TPM) gene expression values across 54 human tissues and sub-tissues from the Genotype-Tissue Expression (GTEx) project version 10^36^. To capture multi-tissue expression patterns, we performed principal component analysis (PCA) on the tissue-normalized expression matrix (each gene’s expression values were z-scored relative to its own expression across tissues) and retained the first five principal components (PCs; PC1-PC5), which together explained 44.9% of the variance in cross-tissue expression profiles.

#### Network proximity features via personalized PageRank

To quantify each target’s network proximity to safety-relevant regions of the HI, we computed personalized PageRank (PPR) scores via multiple seed sets representing gene classes that may confer risk when perturbed (essential, haploinsufficient, cancer-related, and disease-associated genes). A target’s PPR score is its stationary visit probability under a random walk that intermittently restarts to the seed set; thus, it provides a network-based measure of functional proximity to the seed set. We implemented the PPR algorithm via the NetworkX library with a damping factor of *α =0.85*.

We generated PPR scores from six seed sets:

- BAGEL Core-Essential Genes (CEGs): Core essential genes (CEGv2; *n=683*) from Kim and Hart, 2021^18^.
- ClinGen Haploinsufficient Genes: Genes with haploinsufficiency scores of 1–3, indicating any evidence of dosage pathogenicity (*n=552*)^19^.
- IntOGen Pan-cancer Recurrent drivers: Cancer driver genes detected across *\ge 8* cancer types with *\ge100* total samples (*n=72*)^21^.
- IntOGen Activating Broad: Activating oncogenes involved in *\ge 5* cancer types in *\ge 5* cohorts (*n=77*)^21^.
- GenCC strong oncology oncogenes: Oncogenes with classification scores of “Strong” from the GenCC database (*n=121*)^20^.
- DISEASES disease-associated genes: All genes with at least one disease association having a score ≥8.5 from the DISEASES database (*n=41*)^37^.

#### Network centrality features

We calculated five centrality measures to characterize each target’s position in the interactome: betweenness centrality (fraction of shortest paths passing through the node), closeness centrality (inverse of average shortest path length to all other nodes), eigenvector centrality (influence weighted by neighbors’ influence), degree centrality (node degree divided by maximum possible degree), and PageRank centrality (importance based on link structure). Preliminary analyses revealed that all centrality measures were highly correlated and that no combination of centrality measures as features significantly improved performance. We selected betweenness centrality as the only centrality feature for the optimized model.

#### Target biology features

We incorporated target-level features from the Open Targets platform^22^. Specifically, we included all “Target Prioritization factors” except the features “Target in clinic” and “Known safety events” to avoid data leakage caused by including features derived from human clinical data. The included features are as follows:

- Tissue Distribution: categorical scoring of the extent to which a target has detectable expression levels across tissues based on RNA expression data from the Human Protein Atlas^38^
- Tissue Specificity: categorical scoring of the extent to which a target is highly expressed in a small number of tissues based on RNA expression data from the Human Protein Atlas^38^
- Genetic Constraint: loss-of-function observed/expected upper bound fraction (LOEUF) metric, where lower values indicate greater intolerance to loss-of-function variation
- Mouse KO Score: categorical scoring of the severity of the mouse phenotype that results from knockout of the target
- Is In Membrane: binary variable indicating whether the target is located in the cell membrane
- Is Secreted: binary variable indicating whether the target is a secreted protein
- Is Cancer Driver Gene: binary variable indicating whether the target is an oncogene and/or a tumor suppressor gene

Missing values in categorical features were explicitly encoded as “Unknown” rather than imputed.

### Model development and cross-validation

We randomly partitioned the 457 interactome-mapped labeled targets into training (70%, *n=322*) and test (30%, *n=135*) sets via stratified sampling to preserve the toxic-to-safe ratio in both sets. All model selection, feature selection, and hyperparameter optimization were performed exclusively on the training set via cross-validation to prevent data leakage.

For model training, we treated the safety-liability-associated “toxic” label as the positive class. Preliminary testing revealed that gradient boosting classifiers outperformed random forest classifiers and logistic regression models. We performed fivefold stratified cross-validation on the training set, with fold assignments held constant across all experiments to enable direct performance comparisons.

Initial hyperparameter optimization of the gradient boosting classifier was conducted via random search with 5,000 iterations, and the following ranges were evaluated: number of estimators (10–150), maximum tree depth (1–12 and None), minimum samples per leaf (1–10), maximum fraction of features per split (0.0–1.0), and random feature subsets (12–22). Subsequent random searches were performed with iteratively narrowed ranges of hyperparameters. For hyperparameter optimization, 10 random cross-validation splits were used for each evaluated point in the hyperparameter space, and model performance was optimized for the mean area under the receiver operating characteristic curve (ROC AUC) across the 10 cross-validation splits.

### Test set evaluation

After locking the optimal model on the basis of maximal cross-validation ROC AUC performance, we evaluated it once on the held-out test set to assess its generalizability to unseen targets. We report test set performance via the same metrics calculated during cross-validation.

### Pathway enrichment analysis

Pathway enrichment analysis was performed in R via the enrichR package^39^ and the Enrichr KEGG_2021_Human gene-set library to identify KEGG pathways^40^ enriched in the safe and toxic gene sets. Enrichment was conducted separately for each gene set, after which we assessed the overlap between the enriched pathways. To determine whether the two gene sets differed in their underlying biology, we tested for differences in the number of enriched pathways per gene and in the proportion of genes associated with cancer-related pathways.

### Statistical analysis

All analyses other than the pathway enrichment analysis were performed in Python 3.13 via scikit-learn 1.6.1 for machine learning, NumPy 2.2.3 and pandas 2.2.3 for data manipulation, and SciPy 1.15.2 for statistical tests. Network analyses were performed using NetworkX 3.4.2. Statistical significance was assessed at *α =0.05* unless otherwise specified. To account for multiple testing, p-values were adjusted via the Benjamini–Hochberg procedure to control the false discovery rate. These adjusted p-values are reported as q-values.

## Supplementary Information

Supplementary Information.

## Data Availability

The labeled training data used for model development were obtained from Citeline under license and are not publicly available from the authors. Access to this data is subject to Citeline’s licensing terms. All other data were derived from publicly available sources as described in the Methods section. The datasets generated and analyzed during the current study are available from the authors upon reasonable request for academic or non-profit use.

## Code availability

Code used in this study is available from the authors upon reasonable request for academic or non-profit use.
