# Supplementary material for: Target-biology and interactome-derived signatures predict target-level associations with safety-related drug attrition
Source: Sci Rep. 2026 Aug 24;16:25915. doi: 10.1038/s41598-026-65633-y (PMC13503769; doi:10.1038/s41598-026-65633-y)
Supplement: Supplementary file 1 [file 41598_2026_65633_MOESM1_ESM.docx]

**Figure S1: Feature landscape.** Comparison of target feature distributions between targets of widely launched drugs (“Safe”; blue) and safety-liability-associated targets (“Toxic”; red). Widely launched-associated targets were targets annotated for drugs labeled “Widely Launched” in Citeline Pharmaprojects, excluding targets also present in the safety-liability-associated group. Safety-liability-associated targets were targets annotated for drugs in trials labeled “Terminated, Safety/adverse effects” in Citeline Trialtrove, excluding targets also present in the widely launched-associated group. Panels are grouped by feature class, including tissue expression principal components (**A**), tissue expression (**B**), interactome-derived features (**C**), and target-biology features (**D**). Target annotation features are defined in Methods. Briefly, tissue distribution reflects expression breadth across tissues, tissue specificity reflects expression enrichment in a limited number of tissues, genetic constraint is represented by LOEUF, with lower values indicating greater intolerance to loss-of-function variation, and mouse KO score reflects knockout phenotype severity. PPR, personalized PageRank, is a random-walk-with-restart score measuring network proximity to a specified seed set. Feature categories were selected to capture complementary aspects of target biology potentially relevant to safety liability, including tissue context, cross-tissue expression structure, interactome topology, network proximity to biologically important seed sets, genetic constraint, mouse knockout phenotypes, and general target-biology properties. The safety-liability-associated and widely launched-associated target groups differed across multiple independent categories of target-level biology, including tissue expression, interactome proximity to biologically important seed sets, betweenness centrality, genetic constraint, and Open Targets-derived target-biology features. Although individual effect sizes were generally modest, the consistency of these differences across multiple feature classes suggests that the safety-liability-associated labels are not randomly distributed across the interactome. These findings support the use of the labels as operational indicators of target-level safety liability, while not establishing that the annotated target caused the safety-related failure of any individual drug. Tissue-expression differences involving whole blood, spleen, lung, and lymphoid-associated intestinal tissue are interpreted as associations with immune, inflammatory, hematologic, and barrier-tissue biological contexts, not as causal mechanisms for individual drug failures. Abbreviations: KO, knockout; KS test, Kolmogorov–Smirnov test; PC, principal component; TI lymphoid agg., terminal ileum lymphoid aggregate; TPM, transcripts per million. Stars above panels indicate statistical significance, ns for $p>0.05$, * for $p<0.05$, ** for $p<0.01$, *** for $p<0.001$, Kolmogorov‒Smirnov test for panels with empirical CDFs and chi-square test for panels with bar graphs.

**Figure S2: Overlap of PPR seed sets and labeled targets**. **A:** Negative log q-value from the hypergeometric test for significant overlap in nodes for each pairwise comparison of personalized PageRank (PPR) seed sets and labeled target groups used for model development. PPR seed sets and labeled target groups are defined in the Methods. The “Safe” gene set corresponds to targets of drugs labeled as “Widely Launched” in Citeline Pharmaprojects, excluding those that also belong to the Toxic gene set. The “Toxic” gene set corresponds to targets of drugs in trials labeled “Terminated, Safety/adverse effects” in Citeline Trialtrove, excluding those that also belong to the Safe gene set. **B:** UpSet plot of the number of overlapping nodes between the PPR seed sets and labeled target groups. The overlap analysis provides a descriptive assessment of whether widely launched-associated (safe) or safety-liability-associated (toxic) targets are overrepresented in biologically defined interactome neighborhoods. Greater overlap with a seed set suggests that the corresponding target-label group shares network context with that biological gene class. The PPR seed sets were selected based on the prediction that they represented different types of safety-liability regions in the human interactome. Consistent with this prediction, the toxic gene set significantly overlaps with all PPR seed sets. In contrast, the safe gene set significantly overlaps with only the haploinsufficiency gene set (ClinGen Haploinsufficient Genes) and disease-associated gene set (DISEASES All Diseases).

**Table S1**: Statistical comparison of the feature distributions of safe and toxic targets

displayed in Figure S1. For continuous features, the median and interquartile range (IQR) are given for both safe and toxic targets. For categorical features, the modal value is given with the proportion (Prop.) of the group taking the modal value. The p-value is calculated from the Kolmogorov‒Smirnov (KS) test for continuous features and from the chi-square (χ²) test for categorical features. The q-values denote Benjamini‒Hochberg false discovery rate (FDR)-adjusted p-values, with adjustment performed across the set of features reported in this table. Features with $q<0.05$ are deemed significant and highlighted in the table.

| **Target group** | **KEGG pathway** | **Overlapping targets, n** | **Pathway size, n** | **pvalue** | **BH q-value** |
| --- | --- | --- | --- | --- | --- |
| Safe | Neuroactive ligand-receptor interaction | 31 | 341 | 4.89E-28 | 1.13E-25 |
| Safe | Calcium signaling pathway | 17 | 240 | 7.97E-14 | 9.24E-12 |
| Safe | Cocaine addiction | 8 | 49 | 4.87E-10 | 3.77E-08 |
| Safe | Serotonergic synapse | 10 | 113 | 1.53E-09 | 8.86E-08 |
| Safe | Lipid and atherosclerosis | 12 | 215 | 6.67E-09 | 2.71E-07 |
| Safe | cAMP signaling pathway | 12 | 216 | 7.03E-09 | 2.71E-07 |
| Safe | Amphetamine addiction | 8 | 69 | 8.17E-09 | 2.71E-07 |
| Safe | Tyrosine metabolism | 6 | 36 | 6.92E-08 | 2.01E-06 |
| Safe | Pancreatic secretion | 8 | 102 | 1.82E-07 | 4.69E-06 |
| Safe | Dopaminergic synapse | 8 | 132 | 1.32E-06 | 3.05E-05 |
| Safe | Glycosaminoglycan degradation | 4 | 19 | 4.46E-06 | 9.40E-05 |
| Safe | Coronavirus disease | 9 | 232 | 1.12E-05 | 2.16E-04 |
| Safe | Salivary secretion | 6 | 93 | 2.05E-05 | 3.47E-04 |
| Safe | Transcriptional misregulation in cancer | 8 | 192 | 2.09E-05 | 3.47E-04 |
| Safe | cGMP-PKG signaling pathway | 7 | 167 | 6.73E-05 | 9.99E-04 |
| Safe | Thyroid cancer | 4 | 37 | 6.98E-05 | 9.99E-04 |
| Safe | Cytokine-cytokine receptor interaction | 9 | 295 | 7.32E-05 | 9.99E-04 |
| Safe | Influenza A | 7 | 172 | 8.10E-05 | 1.03E-03 |
| Safe | Pathways in cancer | 12 | 531 | 8.48E-05 | 1.03E-03 |
| Safe | Chemical carcinogenesis | 8 | 239 | 9.84E-05 | 1.11E-03 |
| Safe | Leishmaniasis | 5 | 77 | 1.00E-04 | 1.11E-03 |
| Safe | Lysosome | 6 | 128 | 1.23E-04 | 1.25E-03 |
| Safe | Fat digestion and absorption | 4 | 43 | 1.27E-04 | 1.25E-03 |
| Safe | Alcoholism | 7 | 186 | 1.32E-04 | 1.25E-03 |
| Safe | Phenylalanine metabolism | 3 | 17 | 1.35E-04 | 1.25E-03 |
| Safe | Malaria | 4 | 50 | 2.29E-04 | 2.05E-03 |
| Safe | Rheumatoid arthritis | 5 | 93 | 2.44E-04 | 2.10E-03 |
| Safe | PI3K-Akt signaling pathway | 9 | 354 | 2.87E-04 | 2.37E-03 |
| Safe | Circadian entrainment | 5 | 97 | 2.97E-04 | 2.37E-03 |
| Safe | Hematopoietic cell lineage | 5 | 99 | 3.26E-04 | 2.52E-03 |
| Safe | Renin-angiotensin system | 3 | 23 | 3.42E-04 | 2.56E-03 |
| Safe | Chagas disease | 5 | 102 | 3.75E-04 | 2.72E-03 |
| Safe | Th17 cell differentiation | 5 | 107 | 4.67E-04 | 3.28E-03 |
| Safe | Folate biosynthesis | 3 | 26 | 4.95E-04 | 3.38E-03 |
| Safe | Adipocytokine signaling pathway | 4 | 69 | 7.89E-04 | 5.23E-03 |
| Safe | Non-small cell lung cancer | 4 | 72 | 9.26E-04 | 5.97E-03 |
| Safe | Kaposi sarcoma-associated herpesvirus infection | 6 | 193 | 1.10E-03 | 6.88E-03 |
| Safe | Gastric acid secretion | 4 | 76 | 1.13E-03 | 6.92E-03 |
| Safe | Vascular smooth muscle contraction | 5 | 133 | 1.25E-03 | 7.24E-03 |
| Safe | Synaptic vesicle cycle | 4 | 78 | 1.25E-03 | 7.24E-03 |
| Safe | Nicotine addiction | 3 | 40 | 1.77E-03 | 9.90E-03 |
| Safe | Taste transduction | 4 | 86 | 1.79E-03 | 9.90E-03 |
| Safe | MAPK signaling pathway | 7 | 294 | 2.00E-03 | 1.04E-02 |
| Safe | PD-L1 expression and PD-1 checkpoint pathway in cancer | 4 | 89 | 2.03E-03 | 1.04E-02 |
| Safe | Tryptophan metabolism | 3 | 42 | 2.04E-03 | 1.04E-02 |
| Safe | Gastric cancer | 5 | 149 | 2.06E-03 | 1.04E-02 |
| Safe | Pathways of neurodegeneration | 9 | 475 | 2.28E-03 | 1.11E-02 |
| Safe | Small cell lung cancer | 4 | 92 | 2.29E-03 | 1.11E-02 |
| Safe | IL-17 signaling pathway | 4 | 94 | 2.48E-03 | 1.17E-02 |
| Safe | Hepatitis C | 5 | 157 | 2.58E-03 | 1.20E-02 |
| Safe | Necroptosis | 5 | 159 | 2.73E-03 | 1.24E-02 |
| Safe | Hepatitis B | 5 | 162 | 2.96E-03 | 1.29E-02 |
| Safe | JAK-STAT signaling pathway | 5 | 162 | 2.96E-03 | 1.29E-02 |
| Safe | Toll-like receptor signaling pathway | 4 | 104 | 3.57E-03 | 1.53E-02 |
| Safe | Shigellosis | 6 | 246 | 3.71E-03 | 1.56E-02 |
| Safe | Autoimmune thyroid disease | 3 | 53 | 3.97E-03 | 1.64E-02 |
| Safe | Drug metabolism | 4 | 108 | 4.09E-03 | 1.66E-02 |
| Safe | HIF-1 signaling pathway | 4 | 109 | 4.22E-03 | 1.69E-02 |
| Safe | Regulation of lipolysis in adipocytes | 3 | 55 | 4.40E-03 | 1.73E-02 |
| Safe | Nitrogen metabolism | 2 | 17 | 4.58E-03 | 1.77E-02 |
| Safe | NOD-like receptor signaling pathway | 5 | 181 | 4.74E-03 | 1.80E-02 |
| Safe | Cholinergic synapse | 4 | 113 | 4.80E-03 | 1.80E-02 |
| Safe | Other glycan degradation | 2 | 18 | 5.13E-03 | 1.89E-02 |
| Safe | Glycerolipid metabolism | 3 | 61 | 5.89E-03 | 2.13E-02 |
| Safe | Thyroid hormone signaling pathway | 4 | 121 | 6.11E-03 | 2.18E-02 |
| Safe | Steroid biosynthesis | 2 | 20 | 6.32E-03 | 2.22E-02 |
| Safe | Pathogenic Escherichia coli infection | 5 | 197 | 6.74E-03 | 2.33E-02 |
| Safe | Inflammatory bowel disease | 3 | 65 | 7.02E-03 | 2.40E-02 |
| Safe | Osteoclast differentiation | 4 | 127 | 7.23E-03 | 2.43E-02 |
| Safe | Proteoglycans in cancer | 5 | 205 | 7.94E-03 | 2.60E-02 |
| Safe | Mitophagy | 3 | 68 | 7.95E-03 | 2.60E-02 |
| Safe | Renin secretion | 3 | 69 | 8.28E-03 | 2.67E-02 |
| Safe | Rap1 signaling pathway | 5 | 210 | 8.76E-03 | 2.78E-02 |
| Safe | Vitamin digestion and absorption | 2 | 24 | 9.04E-03 | 2.83E-02 |
| Safe | Fluid shear stress and atherosclerosis | 4 | 139 | 9.86E-03 | 3.01E-02 |
| Safe | Measles | 4 | 139 | 9.86E-03 | 3.01E-02 |
| Safe | PPAR signaling pathway | 3 | 74 | 1.00E-02 | 3.02E-02 |
| Safe | Thyroid hormone synthesis | 3 | 75 | 1.04E-02 | 3.09E-02 |
| Safe | Apoptosis | 4 | 142 | 1.06E-02 | 3.11E-02 |
| Safe | Pertussis | 3 | 76 | 1.08E-02 | 3.12E-02 |
| Safe | Collecting duct acid secretion | 2 | 27 | 1.14E-02 | 3.25E-02 |
| Safe | Ras signaling pathway | 5 | 232 | 1.31E-02 | 3.69E-02 |
| Safe | Non-alcoholic fatty liver disease | 4 | 155 | 1.42E-02 | 3.98E-02 |
| Safe | Galactose metabolism | 2 | 31 | 1.48E-02 | 4.09E-02 |
| Safe | Insulin secretion | 3 | 86 | 1.50E-02 | 4.10E-02 |
| Safe | GABAergic synapse | 3 | 89 | 1.65E-02 | 4.44E-02 |
| Safe | Bile secretion | 3 | 90 | 1.70E-02 | 4.52E-02 |
| Safe | Parkinson disease | 5 | 249 | 1.72E-02 | 4.54E-02 |
| Toxic | Pathways in cancer | 38 | 531 | 1.18E-21 | 2.92E-19 |
| Toxic | Toxoplasmosis | 21 | 112 | 5.50E-21 | 6.82E-19 |
| Toxic | Cytokine-cytokine receptor interaction | 28 | 295 | 2.45E-19 | 1.82E-17 |
| Toxic | PI3K-Akt signaling pathway | 30 | 354 | 2.93E-19 | 1.82E-17 |
| Toxic | Apoptosis | 21 | 142 | 9.65E-19 | 4.79E-17 |
| Toxic | Kaposi sarcoma-associated herpesvirus infection | 23 | 193 | 2.91E-18 | 1.20E-16 |
| Toxic | Tuberculosis | 22 | 180 | 9.41E-18 | 3.33E-16 |
| Toxic | Fc gamma R-mediated phagocytosis | 17 | 97 | 1.12E-16 | 3.46E-15 |
| Toxic | AGE-RAGE signaling pathway in diabetic complications | 17 | 100 | 1.91E-16 | 5.27E-15 |
| Toxic | Neutrophil extracellular trap formation | 21 | 189 | 3.88E-16 | 9.62E-15 |
| Toxic | Human T-cell leukemia virus 1 infection | 22 | 219 | 6.41E-16 | 1.43E-14 |
| Toxic | Osteoclast differentiation | 18 | 127 | 6.92E-16 | 1.43E-14 |
| Toxic | Epstein-Barr virus infection | 21 | 202 | 1.51E-15 | 2.89E-14 |
| Toxic | Hepatitis B | 19 | 162 | 3.81E-15 | 6.76E-14 |
| Toxic | NF-kappa B signaling pathway | 16 | 104 | 7.84E-15 | 1.22E-13 |
| Toxic | Toll-like receptor signaling pathway | 16 | 104 | 7.84E-15 | 1.22E-13 |
| Toxic | Human cytomegalovirus infection | 21 | 225 | 1.33E-14 | 1.88E-13 |
| Toxic | PD-L1 expression and PD-1 checkpoint pathway in cancer | 15 | 89 | 1.36E-14 | 1.88E-13 |
| Toxic | TNF signaling pathway | 16 | 112 | 2.62E-14 | 3.42E-13 |
| Toxic | Leishmaniasis | 14 | 77 | 3.51E-14 | 4.35E-13 |
| Toxic | Lipid and atherosclerosis | 20 | 215 | 6.24E-14 | 7.37E-13 |
| Toxic | Chemokine signaling pathway | 19 | 192 | 8.84E-14 | 9.96E-13 |
| Toxic | Acute myeloid leukemia | 13 | 67 | 1.21E-13 | 1.31E-12 |
| Toxic | Influenza A | 18 | 172 | 1.54E-13 | 1.59E-12 |
| Toxic | HIF-1 signaling pathway | 15 | 109 | 3.04E-13 | 3.01E-12 |
| Toxic | Natural killer cell mediated cytotoxicity | 16 | 131 | 3.22E-13 | 3.07E-12 |
| Toxic | VEGF signaling pathway | 12 | 59 | 5.99E-13 | 5.51E-12 |
| Toxic | Pancreatic cancer | 13 | 76 | 6.75E-13 | 5.98E-12 |
| Toxic | JAK-STAT signaling pathway | 17 | 162 | 7.16E-13 | 6.12E-12 |
| Toxic | Chagas disease | 14 | 102 | 1.99E-12 | 1.65E-11 |
| Toxic | T cell receptor signaling pathway | 14 | 104 | 2.62E-12 | 2.09E-11 |
| Toxic | Th17 cell differentiation | 14 | 107 | 3.90E-12 | 3.02E-11 |
| Toxic | Rap1 signaling pathway | 18 | 210 | 4.77E-12 | 3.58E-11 |
| Toxic | Human immunodeficiency virus 1 infection | 18 | 212 | 5.59E-12 | 4.08E-11 |
| Toxic | MicroRNAs in cancer | 21 | 310 | 6.96E-12 | 4.93E-11 |
| Toxic | Small cell lung cancer | 13 | 92 | 8.57E-12 | 5.74E-11 |
| Toxic | Th1 and Th2 cell differentiation | 13 | 92 | 8.57E-12 | 5.74E-11 |
| Toxic | Yersinia infection | 15 | 137 | 9.07E-12 | 5.92E-11 |
| Toxic | Chronic myeloid leukemia | 12 | 76 | 1.43E-11 | 9.09E-11 |
| Toxic | Sphingolipid signaling pathway | 14 | 119 | 1.71E-11 | 1.06E-10 |
| Toxic | Thyroid hormone signaling pathway | 14 | 121 | 2.15E-11 | 1.30E-10 |
| Toxic | C-type lectin receptor signaling pathway | 13 | 104 | 4.22E-11 | 2.49E-10 |
| Toxic | Fc epsilon RI signaling pathway | 11 | 68 | 7.88E-11 | 4.54E-10 |
| Toxic | Salmonella infection | 18 | 249 | 8.18E-11 | 4.61E-10 |
| Toxic | MAPK signaling pathway | 19 | 294 | 1.64E-10 | 9.04E-10 |
| Toxic | Coronavirus disease | 17 | 232 | 2.27E-10 | 1.22E-09 |
| Toxic | Focal adhesion | 16 | 201 | 2.31E-10 | 1.22E-09 |
| Toxic | Proteoglycans in cancer | 16 | 205 | 3.09E-10 | 1.60E-09 |
| Toxic | Chemical carcinogenesis | 17 | 239 | 3.60E-10 | 1.82E-09 |
| Toxic | NOD-like receptor signaling pathway | 15 | 181 | 4.89E-10 | 2.43E-09 |
| Toxic | B cell receptor signaling pathway | 11 | 81 | 5.56E-10 | 2.70E-09 |
| Toxic | Relaxin signaling pathway | 13 | 129 | 6.52E-10 | 3.11E-09 |
| Toxic | Type II diabetes mellitus | 9 | 46 | 7.30E-10 | 3.41E-09 |
| Toxic | ErbB signaling pathway | 11 | 85 | 9.44E-10 | 4.33E-09 |
| Toxic | Insulin resistance | 12 | 108 | 9.70E-10 | 4.37E-09 |
| Toxic | Insulin signaling pathway | 13 | 137 | 1.38E-09 | 6.11E-09 |
| Toxic | Cholinergic synapse | 12 | 113 | 1.64E-09 | 7.16E-09 |
| Toxic | Ras signaling pathway | 16 | 232 | 1.90E-09 | 8.10E-09 |
| Toxic | Prolactin signaling pathway | 10 | 70 | 2.07E-09 | 8.70E-09 |
| Toxic | Rheumatoid arthritis | 11 | 93 | 2.51E-09 | 1.04E-08 |
| Toxic | Non-small cell lung cancer | 10 | 72 | 2.75E-09 | 1.12E-08 |
| Toxic | Neurotrophin signaling pathway | 12 | 119 | 3.00E-09 | 1.20E-08 |
| Toxic | Gastric cancer | 13 | 149 | 3.88E-09 | 1.53E-08 |
| Toxic | Prostate cancer | 11 | 97 | 3.96E-09 | 1.53E-08 |
| Toxic | Shigellosis | 16 | 246 | 4.41E-09 | 1.68E-08 |
| Toxic | Platelet activation | 12 | 124 | 4.81E-09 | 1.81E-08 |
| Toxic | Amoebiasis | 11 | 102 | 6.79E-09 | 2.51E-08 |
| Toxic | FoxO signaling pathway | 12 | 131 | 9.01E-09 | 3.29E-08 |
| Toxic | Colorectal cancer | 10 | 86 | 1.61E-08 | 5.77E-08 |
| Toxic | Hepatocellular carcinoma | 13 | 168 | 1.66E-08 | 5.89E-08 |
| Toxic | Measles | 12 | 139 | 1.76E-08 | 6.16E-08 |
| Toxic | Signaling pathways regulating pluripotency of stem cells | 12 | 143 | 2.43E-08 | 8.36E-08 |
| Toxic | Phospholipase D signaling pathway | 12 | 148 | 3.57E-08 | 1.21E-07 |
| Toxic | IL-17 signaling pathway | 10 | 94 | 3.82E-08 | 1.28E-07 |
| Toxic | Human papillomavirus infection | 17 | 331 | 4.78E-08 | 1.58E-07 |
| Toxic | Inflammatory mediator regulation of TRP channels | 10 | 98 | 5.72E-08 | 1.87E-07 |
| Toxic | Cellular senescence | 12 | 156 | 6.41E-08 | 2.05E-07 |
| Toxic | Glioma | 9 | 75 | 6.46E-08 | 2.05E-07 |
| Toxic | Hepatitis C | 12 | 157 | 6.88E-08 | 2.13E-07 |
| Toxic | Progesterone-mediated oocyte maturation | 10 | 100 | 6.95E-08 | 2.13E-07 |
| Toxic | Viral protein interaction with cytokine and cytokine receptor | 10 | 100 | 6.95E-08 | 2.13E-07 |
| Toxic | African trypanosomiasis | 7 | 37 | 7.69E-08 | 2.33E-07 |
| Toxic | Autophagy | 11 | 137 | 1.48E-07 | 4.43E-07 |
| Toxic | Viral carcinogenesis | 13 | 203 | 1.56E-07 | 4.59E-07 |
| Toxic | Fluid shear stress and atherosclerosis | 11 | 139 | 1.72E-07 | 5.02E-07 |
| Toxic | GnRH secretion | 8 | 64 | 2.57E-07 | 7.41E-07 |
| Toxic | Gap junction | 9 | 88 | 2.63E-07 | 7.48E-07 |
| Toxic | Inflammatory bowel disease | 8 | 65 | 2.91E-07 | 8.19E-07 |
| Toxic | Growth hormone synthesis, secretion and action | 10 | 119 | 3.62E-07 | 1.01E-06 |
| Toxic | AMPK signaling pathway | 10 | 120 | 3.91E-07 | 1.08E-06 |
| Toxic | Neuroactive ligand-receptor interaction | 16 | 341 | 4.08E-07 | 1.11E-06 |
| Toxic | GnRH signaling pathway | 9 | 93 | 4.23E-07 | 1.14E-06 |
| Toxic | Renal cell carcinoma | 8 | 69 | 4.65E-07 | 1.24E-06 |
| Toxic | mTOR signaling pathway | 11 | 154 | 4.85E-07 | 1.28E-06 |
| Toxic | Staphylococcus aureus infection | 9 | 95 | 5.08E-07 | 1.33E-06 |
| Toxic | Choline metabolism in cancer | 9 | 98 | 6.63E-07 | 1.71E-06 |
| Toxic | Necroptosis | 11 | 159 | 6.67E-07 | 1.71E-06 |
| Toxic | Herpes simplex virus 1 infection | 19 | 498 | 7.76E-07 | 1.96E-06 |
| Toxic | Vascular smooth muscle contraction | 10 | 133 | 1.02E-06 | 2.54E-06 |
| Toxic | Estrogen signaling pathway | 10 | 137 | 1.33E-06 | 3.30E-06 |
| Toxic | Aldosterone-regulated sodium reabsorption | 6 | 37 | 1.76E-06 | 4.33E-06 |
| Toxic | Endometrial cancer | 7 | 58 | 1.88E-06 | 4.57E-06 |
| Toxic | Long-term depression | 7 | 60 | 2.37E-06 | 5.66E-06 |
| Toxic | Viral myocarditis | 7 | 60 | 2.37E-06 | 5.66E-06 |
| Toxic | Breast cancer | 10 | 147 | 2.53E-06 | 5.97E-06 |
| Toxic | Cell adhesion molecules | 10 | 148 | 2.69E-06 | 6.29E-06 |
| Toxic | Oxytocin signaling pathway | 10 | 154 | 3.84E-06 | 8.91E-06 |
| Toxic | Transcriptional misregulation in cancer | 11 | 192 | 4.23E-06 | 9.72E-06 |
| Toxic | Long-term potentiation | 7 | 67 | 5.03E-06 | 1.14E-05 |
| Toxic | Alzheimer disease | 15 | 369 | 5.53E-06 | 1.25E-05 |
| Toxic | Central carbon metabolism in cancer | 7 | 70 | 6.76E-06 | 1.51E-05 |
| Toxic | Hematopoietic cell lineage | 8 | 99 | 7.35E-06 | 1.63E-05 |
| Toxic | Melanoma | 7 | 72 | 8.17E-06 | 1.79E-05 |
| Toxic | Intestinal immune network for IgA production | 6 | 48 | 8.49E-06 | 1.85E-05 |
| Toxic | p53 signaling pathway | 7 | 73 | 8.96E-06 | 1.93E-05 |
| Toxic | Apelin signaling pathway | 9 | 137 | 1.08E-05 | 2.29E-05 |
| Toxic | Malaria | 6 | 50 | 1.08E-05 | 2.29E-05 |
| Toxic | Pertussis | 7 | 76 | 1.17E-05 | 2.46E-05 |
| Toxic | Parathyroid hormone synthesis, secretion and action | 8 | 106 | 1.22E-05 | 2.54E-05 |
| Toxic | Leukocyte transendothelial migration | 8 | 114 | 2.08E-05 | 4.29E-05 |
| Toxic | Pathways of neurodegeneration | 16 | 475 | 2.78E-05 | 5.70E-05 |
| Toxic | Pathogenic Escherichia coli infection | 10 | 197 | 3.31E-05 | 6.73E-05 |
| Toxic | Calcium signaling pathway | 11 | 240 | 3.43E-05 | 6.92E-05 |
| Toxic | Allograft rejection | 5 | 38 | 3.83E-05 | 7.60E-05 |
| Toxic | Primary immunodeficiency | 5 | 38 | 3.83E-05 | 7.60E-05 |
| Toxic | TGF-beta signaling pathway | 7 | 94 | 4.71E-05 | 9.26E-05 |
| Toxic | Bladder cancer | 5 | 41 | 5.58E-05 | 1.09E-04 |
| Toxic | Systemic lupus erythematosus | 8 | 135 | 6.98E-05 | 1.35E-04 |
| Toxic | Regulation of actin cytoskeleton | 10 | 218 | 7.77E-05 | 1.49E-04 |
| Toxic | Longevity regulating pathway | 7 | 102 | 7.93E-05 | 1.51E-04 |
| Toxic | Axon guidance | 9 | 182 | 1.00E-04 | 1.90E-04 |
| Toxic | Carbohydrate digestion and absorption | 5 | 47 | 1.09E-04 | 2.04E-04 |
| Toxic | Adrenergic signaling in cardiomyocytes | 8 | 150 | 1.46E-04 | 2.72E-04 |
| Toxic | Non-alcoholic fatty liver disease | 8 | 155 | 1.83E-04 | 3.38E-04 |
| Toxic | Autoimmune thyroid disease | 5 | 53 | 1.94E-04 | 3.56E-04 |
| Toxic | Diabetic cardiomyopathy | 9 | 203 | 2.29E-04 | 4.17E-04 |
| Toxic | cGMP-PKG signaling pathway | 8 | 167 | 3.03E-04 | 5.49E-04 |
| Toxic | Circadian entrainment | 6 | 97 | 4.54E-04 | 8.16E-04 |
| Toxic | Prion disease | 10 | 273 | 4.77E-04 | 8.51E-04 |
| Toxic | Adipocytokine signaling pathway | 5 | 69 | 6.66E-04 | 1.17E-03 |
| Toxic | Amphetamine addiction | 5 | 69 | 6.66E-04 | 1.17E-03 |
| Toxic | RIG-I-like receptor signaling pathway | 5 | 70 | 7.11E-04 | 1.24E-03 |
| Toxic | Glucagon signaling pathway | 6 | 107 | 7.64E-04 | 1.33E-03 |
| Toxic | Retrograde endocannabinoid signaling | 7 | 148 | 7.78E-04 | 1.34E-03 |
| Toxic | Bacterial invasion of epithelial cells | 5 | 77 | 1.10E-03 | 1.88E-03 |
| Toxic | Cholesterol metabolism | 4 | 50 | 1.62E-03 | 2.75E-03 |
| Toxic | Cell cycle | 6 | 124 | 1.64E-03 | 2.77E-03 |
| Toxic | Oocyte meiosis | 6 | 129 | 2.00E-03 | 3.36E-03 |
| Toxic | Salivary secretion | 5 | 93 | 2.53E-03 | 4.22E-03 |
| Toxic | Notch signaling pathway | 4 | 59 | 2.98E-03 | 4.93E-03 |
| Toxic | Melanogenesis | 5 | 101 | 3.62E-03 | 5.94E-03 |
| Toxic | Asthma | 3 | 31 | 3.70E-03 | 6.04E-03 |
| Toxic | Phagosome | 6 | 152 | 4.52E-03 | 7.32E-03 |
| Toxic | Nicotinate and nicotinamide metabolism | 3 | 35 | 5.24E-03 | 8.43E-03 |
| Toxic | Adherens junction | 4 | 71 | 5.79E-03 | 9.26E-03 |
| Toxic | Serotonergic synapse | 5 | 113 | 5.82E-03 | 9.26E-03 |
| Toxic | Glutamatergic synapse | 5 | 114 | 6.04E-03 | 9.54E-03 |
| Toxic | Thyroid cancer | 3 | 37 | 6.13E-03 | 9.62E-03 |
| Toxic | Type I diabetes mellitus | 3 | 43 | 9.31E-03 | 1.45E-02 |
| Toxic | Purine metabolism | 5 | 129 | 1.00E-02 | 1.56E-02 |
| Toxic | Insulin secretion | 4 | 86 | 1.13E-02 | 1.74E-02 |
| Toxic | Spinocerebellar ataxia | 5 | 143 | 1.51E-02 | 2.32E-02 |
| Toxic | Endocrine and other factor-regulated calcium reabsorption | 3 | 53 | 1.64E-02 | 2.50E-02 |
| Toxic | Phosphatidylinositol signaling system | 4 | 97 | 1.69E-02 | 2.55E-02 |
| Toxic | Aldosterone synthesis and secretion | 4 | 98 | 1.75E-02 | 2.63E-02 |
| Toxic | Regulation of lipolysis in adipocytes | 3 | 55 | 1.81E-02 | 2.71E-02 |
| Toxic | Pyrimidine metabolism | 3 | 56 | 1.90E-02 | 2.82E-02 |
| Toxic | Legionellosis | 3 | 57 | 1.99E-02 | 2.94E-02 |
| Toxic | Arginine biosynthesis | 2 | 22 | 2.05E-02 | 3.01E-02 |
| Toxic | Cushing syndrome | 5 | 155 | 2.07E-02 | 3.02E-02 |
| Toxic | cAMP signaling pathway | 6 | 216 | 2.27E-02 | 3.29E-02 |
| Toxic | Cytosolic DNA-sensing pathway | 3 | 63 | 2.59E-02 | 3.73E-02 |
| Toxic | Maturity onset diabetes of the young | 2 | 26 | 2.81E-02 | 4.03E-02 |
| Toxic | Glycolysis / Gluconeogenesis | 3 | 67 | 3.03E-02 | 4.32E-02 |
| Toxic | Mitophagy | 3 | 68 | 3.15E-02 | 4.46E-02 |
| Toxic | Epithelial cell signaling in Helicobacter pylori infection | 3 | 70 | 3.39E-02 | 4.78E-02 |

**Table S2: Statistically significant KEGG pathway enrichment results for labeled target groups.** Pathway enrichment used the KEGG_2021_Human gene-set library accessed through Enrichr^40,41^. The table lists KEGG pathways significantly enriched among targets in each labeled target group. Target group indicates the simplified operational label group tested: “Safe” for widely launched-associated targets and “Toxic” for safety-liability-associated targets. Overlapping targets, n indicates the number of targets from the corresponding group that overlap the KEGG pathway, and Pathway size, n indicates the total number of genes in the KEGG pathway background. P values are nominal enrichment p-values from the pathway enrichment test. BH q-values are Benjamini-Hochberg false-discovery-rate-adjusted p-values. Only pathways meeting the statistical significance threshold after Benjamini-Hochberg adjustment are shown.
